# A murine model of cerebral cavernous malformations with acute hemorrhage

**DOI:** 10.1016/j.isci.2022.103943

**Published:** 2022-02-18

**Authors:** Claudio Maderna, Federica Pisati, Claudio Tripodo, Elisabetta Dejana, Matteo Malinverno

**Affiliations:** 1 Vascular Biology Unit, The FIRC Institute of Molecular Oncology Foundation, Milan 20139, Italy; 2 Tumour and Microenvironment Histopathology Unit, FIRC Institute of Molecular Oncology (IFOM), Milan, Italy; 3 Tumour Immunology Unit, University of Palermo, Palermo, Italy; 4 Department of Immunology, Genetics and Pathology, Uppsala University, Uppsala 752 37, Sweden

**Keywords:** Vascular remodeling, Developmental neuroscience, Model organism

## Abstract

Cavernomas are multi-lumen and blood-filled vascular malformations which form in the brain and the spinal cord. They lead to hemorrhage, epileptic seizures, neurological deficits, and paresthesia. An effective medical treatment is still lacking, and the available murine models for cavernomas have several limitations for preclinical studies. These include disease phenotypes that differ from human diseases, such as restriction of the lesions to the cerebellum, and absence of acute hemorrhage. Additional limitations of current murine models include rapid development of lesions, which are lethal before the first month of age. Here, we have characterized a murine model that recapitulates features of the human disease: lesions develop after weaning throughout the entire CNS, including the spinal cord, and undergo acute hemorrhage. This provides a preclinical model to develop new drugs for treatment of acute hemorrhage in the brain and spinal cord, as an unmet medical emergency for patients with cavernomas.

## Highlights

*Ccm3* deletion in endothelial progenitors drives cavernoma formation in a mouse model
• Mice develop acute hemorrhage and inflammation in brain and spinal cord
• The spleen has increased vascular density and altered hemopoiesis
• This model represents a useful tool for mechanistic studies and drug screening

## Graphical abstract

**Figure:**
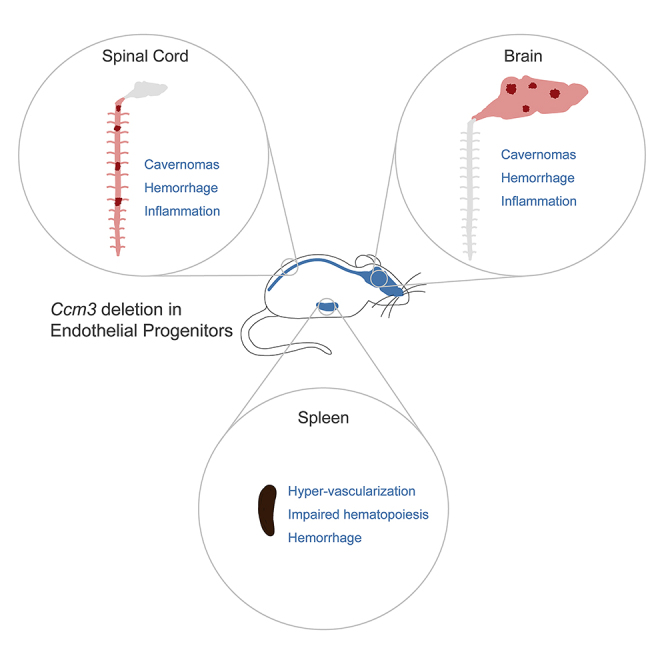

## Introduction

Cerebral cavernous malformations (CCMs) are also known as cerebral cavernomas or cavernous angiomas. These are clusters of capillary–venous malformations that are mainly found in the CNS. CCMs occur in sporadic and familial forms, and they have an overall prevalence of 0.1%–0.5% (Otten et al., 1989; Morris et al., 2009; Al-Holou et al., 2012). Although familial forms of CCM develop multiple cavernomas that increase in size and number throughout life, the sporadic form is usually characterized by single lesions that appear at an adult age.

Familial cases follow autosomal-dominant inheritance because of loss-of-function mutations in one of the three genes known as *CCM1*/*KRIT1*, *CCM2*/*malcavernin*, and *CCM3*/*PDCD10*. These encode three cytoplasmic proteins, and as well as their other specific functions, these form a tripartite complex that is associated with endothelial cell junctions, to promote increased junction strength and maintain endothelial homeostasis (Lampugnani et al., 2017; Valentino et al., 2020).

Despite their different clinical presentations and genetic backgrounds, CCMs share similar characteristics and symptomatology. They appear as dilated and multi-lumen vessels that are lined with a single layer of endothelial cells and are devoid of surrounding parenchymal cells; this makes them fragile and prone to rupture. Indeed, the major clinical symptoms are related to intracerebral hemorrhage (25%), which results in cavernoma-related epileptic seizures (50%), focal neurological deficits (25%), and nonspecific headache.

The estimated 5-year risk for the first intracerebral hemorrhage for patients with CCM is 3%–8%, which depends on the location, whereby the brainstem has the highest probability for bleeding. This increases to up to 30% for patients with CCM that have already shown bleeding. The risk to develop cavernoma-related epileptic seizures varies between 1.5 and 2.4% per patient year, with the risk of recurrent seizures at around 94%. Although CCM hemorrhages tend to be intracerebral and of low volume, the case fatalities described in the literature range from 0% to 17% for recurrent intracerebral hemorrhage in brainstem CCMs (Bradac and Benes, 2020; Li et al., 2021a, 2021b). Spinal cord cavernous malformations are usually considered to be less common than CCMs but recent studies estimated a prevalence of these lesions of between 23 and 72% in patients with familial CCMs (Cohen-Gadol et al., 2006; Toldo et al., 2009; de Vos et al., 2017; Mabray et al., 2020). Major symptoms associated with spinal cord cavernous malformations include muscular weakness (60.5%), tingling and numbness (57.8%), pain (33.8%), bladder and/or bowel impairment (23.6%), and respiratory distress (0.5%), with an annual hemorrhage rate of 2.1% (Badhiwala et al., 2014).

Emerging evidence indicates that inflammation and immune responses are also involved in the pathogenesis of CCM, and that these have crucial roles in determining the severity of the disease. T cells, B cells, and macrophages have been shown to accumulate within lesions, where they positively correlate with the severity of the lesion burden and hemorrhage in patients (Sanus et al., 2007; Shi et al., 2009; Choquet et al., 2014), whereas B-cell depletion can reduce disease progression in a murine model of CCM (Shi et al., 2016). Compounds with immunosuppressant and/or anti-inflammatory activities have been used successfully to reduce the severity of CCM induced in mice (Bravi et al., 2015; Tang et al., 2017, 2019). Consistent with a role of inflammation in CCM, several cytokines that circulate in the peripheral blood have been identified as plasma inflammatory biomarkers that reflect seizures and recent hemorrhagic activity in patients with CCM (Girard et al., 2018a; 2018b).

At present, there are no drugs available to prevent and/or reduce the clinical symptoms of CCM, and the only available curative therapy is surgical resection of the CCM. However, even after decades of experimentation, the risk/benefit balance of this approach remains conflicting; some studies report worsening of symptoms after surgery approximately in 15% of cases and mortality in 1.5% of cases. Therefore, the absence of effective pharmacological therapies to treat CCM demonstrate an unmet medical need, and especially for those patients with cavernomas that cannot be safely resected.

Great efforts have been made over the last two decades to define the molecular and cellular mechanisms that underlie CCM. We now know that CCM lesions arise from a few resident endothelial progenitors that undergo uncontrolled clonal expansion in response to loss of the *CCM* genes. These cells can attract healthy neighboring cells, which then acquire pathological features and participate in the growth of the cavernoma (Detter et al., 2018; Malinverno et al., 2019). Both mutated and wild-type cells that line the cavernoma share the same pathological features, which are characterized by increased MEKK3-MEK5-ERK5-KLF2/4(Cuttano et al., 2016; Zhou et al., 2016) and TGFβ/BMPs (Maddaluno et al., 2013; Bravi et al., 2015) signaling pathways, which leads to the so-called endothelial-to-mesenchymal transition. Moreover, contributions to the complex clinical manifestation of CCM include activated β-catenin (Bravi et al., 2015), Rho/ROCK (Crose et al., 2009; Whitehead et al., 2009), and Angpt2/Tie2(Jenny Zhou et al., 2016; Zhou et al., 2021) signaling pathways, increased angiogenesis (Lopez-Ramirez et al., 2017), and an altered microbiome (Tang et al., 2017).

Several murine models of CCM have been generated that were fundamental for insight into the molecular cues of CCM and to test the efficacies of different drugs to ameliorate the lesion burden. However, most of these murine models of CCM develop lesions very quickly after birth and are lethal before or near the first month of age. This represents a big limitation when testing the efficacies of drugs to reduce established lesions. Other limitations include the spatial restriction of most lesions to the cerebellum, and to the absence of spontaneous acute hemorrhage and inflammatory infiltrates.

We previously generated a murine model in which the deletion of the *Ccm3* gene was restricted to *Procr*-positive endothelial progenitor cells (EPCs) (Malinverno et al., 2019). These mice developed lesions that resembled human pathology, and showed increased expression of *KLF4* and endothelial-to-mesenchymal transition markers.

Here, we further characterized this model and show that it develops lesions with slow progression from 1 month to 6 months of age, when *Ccm3* is deleted at early postnatal stages. These lesions are not restricted to the cerebellum, but instead spread throughout the brain and the spinal cord, and they develop acute and chronic hemorrhage and inflammatory cell infiltration. We also report on spleen red pulp hypervascularization, which is associated with partial regression of the white pulp lymphoid components and an imbalanced splenic hematopoiesis that is shifted toward the erythroid lineage, to the detriment of the myeloid populations.

This murine model represents a useful preclinical tool to test drugs with a particular focus on regression or stabilization of existing cavernomas, which are prone to bleeding or that have already shown bleeding.

## Results

### Endothelial-progenitor-cell-specific deletion of *Ccm3* induces progressive formation of CCM lesions throughout the brain

**Figure 1:**
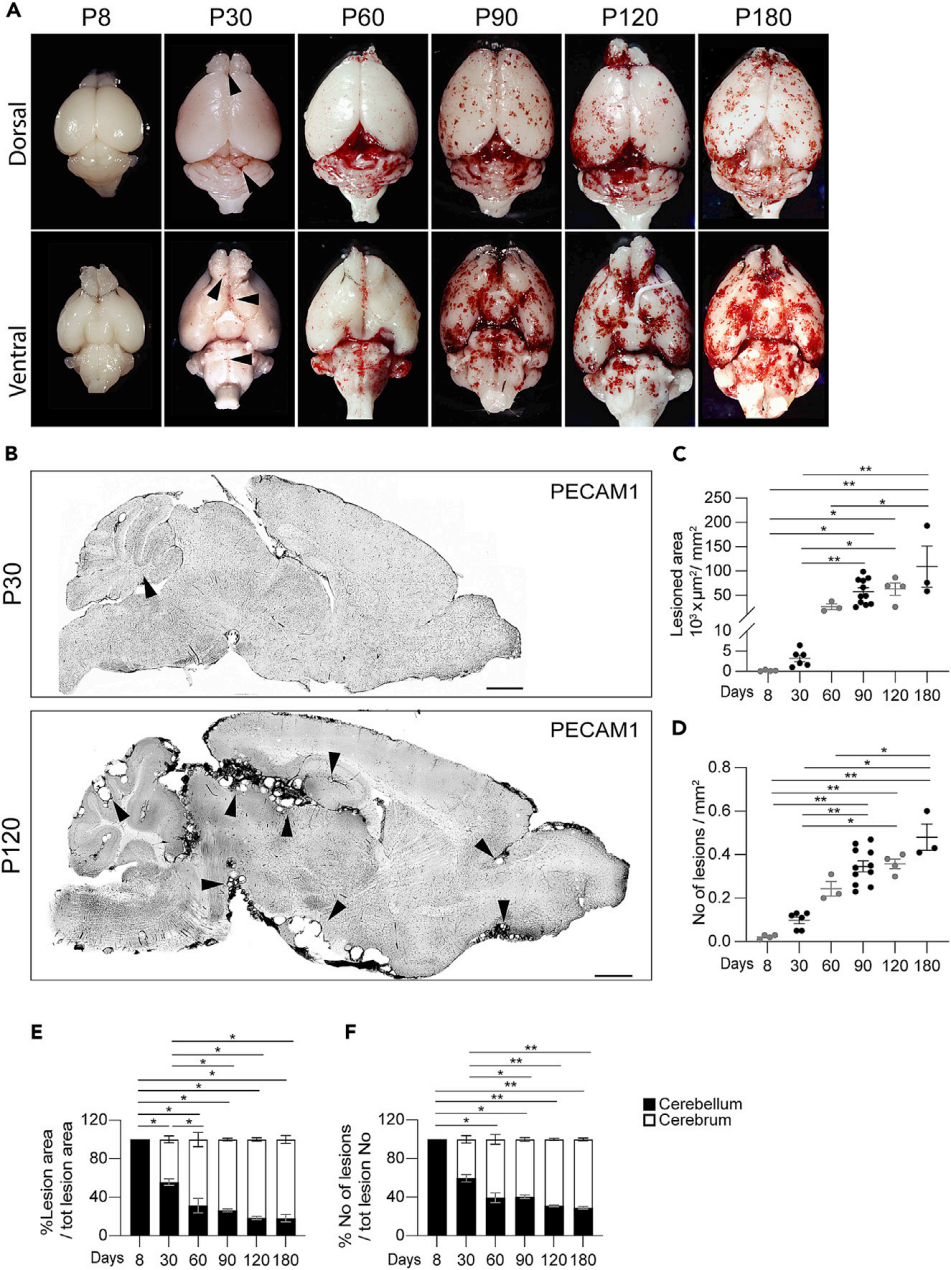
Endothelial-progenitor-cells-specific deletion of *Ccm3* results in progressive formation of lesions throughout the brain *Procr*^CreERT2−IRES-tdTomato/+/^*Ccm3*^f/f^ mice received a single dose of tamoxifen at P1 and were analyzed at the indicated times. (A and B) Representative photographs of whole brains (A) and tiling of brain sections (B) stained for PECAM1, showing lesion burden and distribution. Black arrowheads, lesions. Scale bars: 1000 μm. (C) Quantification of total lesioned area. Data are means ± SE. Each dot represents an animal; p < 0.0001 among groups (ANOVA); ∗p < 0.05, ∗∗p < 0.01 (Tukey’s *post hoc* tests). (D) Quantification of lesion numbers. Data are means ± SE. Each dot represents an animal; p < 0.001 among groups (Kruskal-Wallis tests); ∗p < 0.05, ∗∗p < 0.01 (Dunn’s *post hoc* tests). (E) Quantification of distribution of lesion area between cerebellum and cerebrum. Data are means ± SE. Each dot represents an animal; p < 0.001 among groups (ANOVA) ∗p < 0.00 1 (Tukey’s post hoc tests). (F) Quantification of distribution of lesion numbers between cerebellum and cerebrum. Data are means ± SE. Each dot represents an animal; p < 0.001 among groups (Kruskal-Wallis tests) ∗p < 0.05, ∗∗p < 0.01 (Dunn’s *post hoc* tests).

We have previously generated a murine model for the deletion of the *Ccm3* gene specifically in the *Procr*-positive endothelial progenitor cells (EPC) (Malinverno et al., 2019). This model combines the *Ccm3* gene floxed (*Ccm3*^f/f^) with the inducible form of the Cre recombinase driven by the *Procr* promoter (*Procr*^CreERT2−IRES-tdTomato/+^). These animals (named *Ccm3*^EPCKO^) developed cavernous malformations in the brain and retina which recapitulated the pathogenetic features described in other murine models, where the *Ccm3* gene was inactivated in all the endothelial cells (*Ccm3*^ECKO^). Lesions were composed of mutant and wild-type cells that overexpressed *KLF4* and endothelial-to-mesenchymal transition genes. A single dose of tamoxifen on the day of birth (P1) resulted in full penetrance of the pathological phenotype, which started appearing macroscopically around 1 month of age, and which became more severe after 2 months–3 months (Figure 1A).

Histological analysis revealed that a few small lesions developed as early as P8, and then increased in number and size up to 6 months of age (Figures 1B–1D). The lesions developed rapidly between 1 month and 3 months of age, when they reached a sort of plateau, and increased slowly up to 6 months. One 6-month-old mouse developed a mass in the cortex, which was identified as a giant cavernoma by histopathological analysis (Figure S1).

Although the lesions were mainly restricted to the cerebellum at P8, they started to form in the rest of the brain over time; after 3 months, 70%–75% of the lesioned area was located in the cerebrum (Figures 1E, 1F, and S2). This is in line with the lesion distribution in familial patients, which was reported to be around 70%–80% in the cerebrum (Brunereau et al., 2000; Labauge et al., 2001; Dalyai et al., 2011).

We confirmed also that endothelial cells lining the cavernomas in *Ccm3*^EPCKO^ mice showed evidence of endothelial-to-mesenchymal transition, in a way that is comparable to what has been shown in other models where *Ccm3* is deleted in all ECs. ECs in lesions of *Ccm3*^EPCKO^ mice expressed higher levels of KLF4 and of a set EndMT markers, i.e., SCA1, FN1, and αSMA, if compared to ECs of surrounding normal vessels, thus resembling the expression pattern of the same proteins in *Ccm3*^ECKO^ mice (Figure S3).

A delay in the age of tamoxifen administration (i.e., at P2 or P3) resulted in slower progression of the disease and much milder phenotype (Figure S4), whereas no phenotype was observed in animals which received tamoxifen at adult age. This is in agreement with what has been described for other murine models of CCM (Boulday et al., 2011; Detter et al., 2020; Ren et al., 2021; Zhou et al., 2021).

### Late-stage lesions develop inflammatory infiltrate and acute hemorrhage

**Figure 2:**
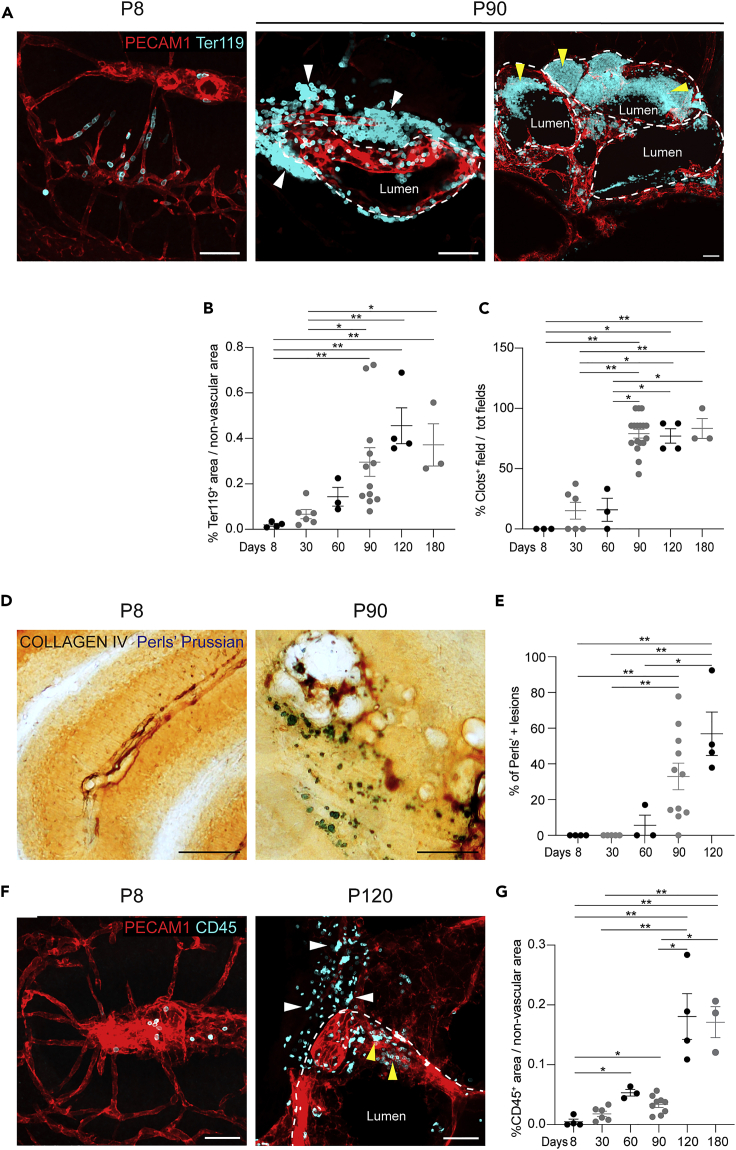
Late-stage lesions develop acute and chronic hemorrhages and inflammatory cell infiltrates Representative brain sections and relative quantification showing acute and chronic hemorrhages and inflammatory infiltrates. Scale bars: 50 μm. (A) Representative brain sections at P8 and P90 stained for PECAM1 and Ter119. White arrowheads (middle), extravasated erythrocytes; yellow arrowheads (right), Ter119-positive clots within the lumen of large cavernomas. (B) Quantification of erythrocyte extravasation. Data are means ± SE. Each dot represents an animal; p < 0.001 among groups (Kruskal-Wallis tests); ∗p < 0.05, ∗∗p < 0.01 (Dunn’s *post hoc* tests). (C) Quantification of clots in brain sections. Data are means ± SE. Each dot represents an animal; p < 0.0005 among groups (Kruskal-Wallis tests); ∗p < 0.05, ∗∗p < 0.01 (Dunn’s *post hoc* tests). (D) Representative brain sections showing chronic hemorrhage, visualized with Perls' Prussian blue stain. Vessels counterstained for collagen IV. (E) Quantification of chronic hemorrhage. Data are means ± SE. Each dot represents an animal; p < 0.002 among groups (Kruskal-Wallis tests); ∗p < 0.05 (Dunn’s *post hoc* tests). (F) Representative brain sections stained for PECAM1 and CD45 showing leukocyte infiltration. White arrowheads, extravasated CD45-positive cells; yellow arrowheads, cells within the lumen. (G) Quantification of leukocyte extravasation. Data are means ± SE. Each dot represents an animal; p < 0.001 among groups (Kruskal-Wallis tests); ∗p < 0.02, ∗∗p < 0.001 (Dunn’s *post hoc* tests).

At the later stages, human CCM is characterized by cerebral hemorrhage, which causes the majority of clinical symptoms. In particular, acute hemorrhage is the cause of most of the more severe symptoms, such as epileptic seizures and focal neurological deficits. In the model described here, the mice showed brain hemorrhages in proximity to the cavernomas, which were indicated by massive extravasation of red blood cells stained by Ter119 (Figures 2A and 2B). These acute hemorrhages occurred quite early, and then dramatically increased over time, up to 4 months of age. Ter119 staining also revealed the formation of blood clots in the lumen of late-stage cavernomas, as mainly after 3 months of age (Figures 2A and 2C). Whether the formation of blood clots in the lesions helps to reduce bleeding-related symptoms, or on the contrary, participates in worsening the severity of the disease, has still to be determined. In parallel, we provide evidence of chronic hemorrhage as revealed using Perls' Prussian blue dye, which stained the nonheme iron that surrounds these cavernomas from two months of age (Figures 2D and 2E).

Another important aspect of the pathogenesis of human CCM is inflammation, which has been shown to correlate with severity of hemorrhage and lesion burden (Sanus et al., 2007; Shi et al., 2009; Choquet et al., 2014). By staining for CD45, a pan-leukocyte protein, we revealed infiltration of leukocytes in the parenchyma surrounding the cavernomas in 4-month-old mice, which supported a concomitant local inflammatory response (Figures 2F and 2G).

### The spinal cord develops CCM lesions that resemble the features of brain lesions

**Figure 3:**
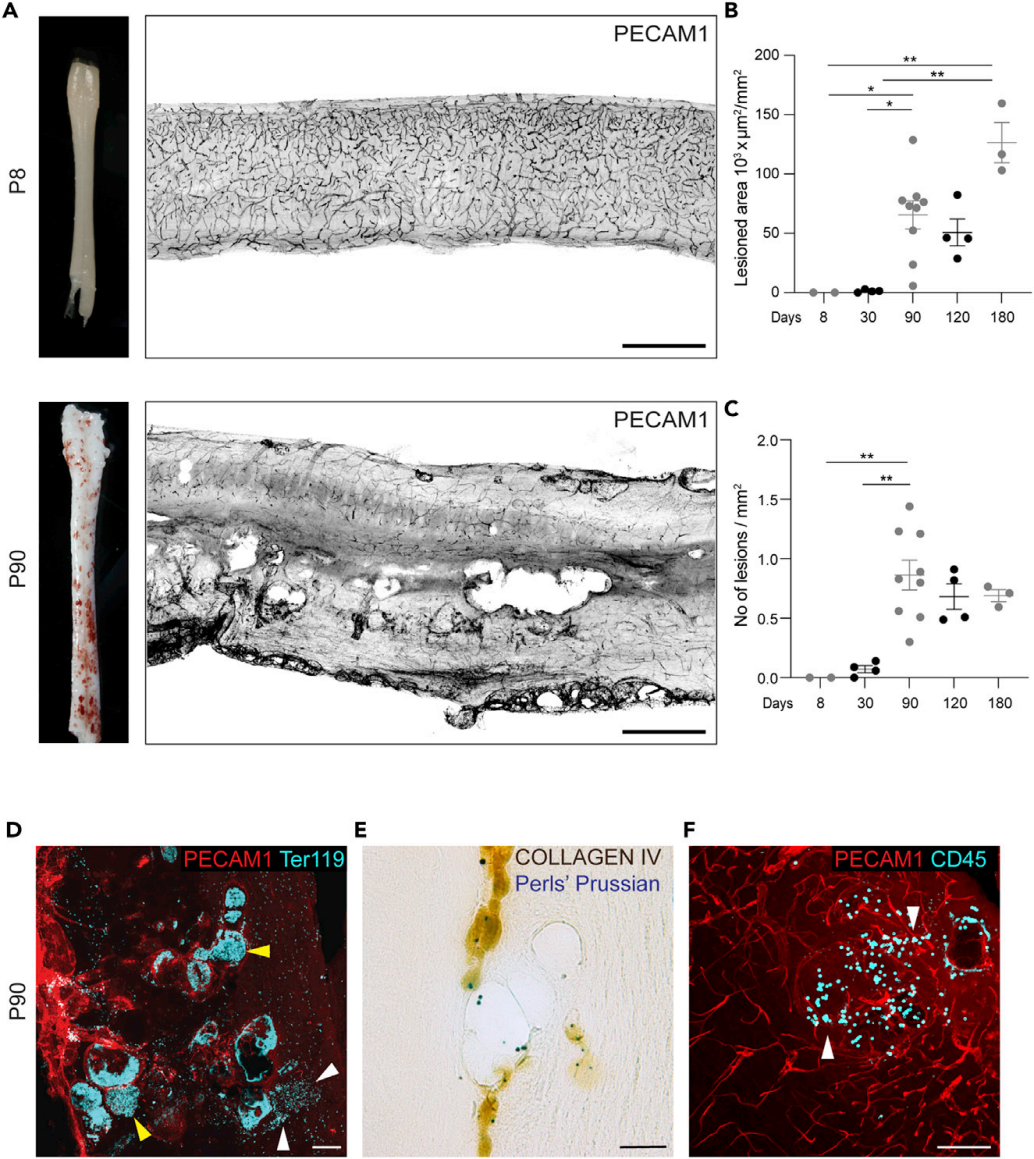
The spinal cord develops CCM lesions with hemorrhages and inflammatory infiltrates (A) Representative photographs of whole spinal cord and tiling of sagittal sections stained for PECAM1, showing lesion burden and distribution. (B) Quantification of total lesioned area. Data are means ± SE. Each dot represents an animal; p < 0.005 among groups (Kruskal-Wallis tests); ∗p < 0.05, ∗∗p < 0.01 (Dunn’s *post hoc* tests). (C) Quantification of lesion numbers. Data are means ± SE. Each dot represents an animal; p < 0.01 among groups (ANOVA); ∗∗p < 0.01 (Tukey’s *post hoc* tests). (D–F) Representative brain sections showing acute (D) and chronic (E) hemorrhages and inflammatory cell infiltrates (F). Scale bar: 500 μm (A); 100 μm (D, F); 50 μm (E). White arrowheads, extravasated cells; yellow arrowheads, clots.

Cavernous malformations located in the spinal cord are estimated to affect 23%–72% of patients with the familial form of CCMs (Cohen-Gadol et al., 2006; Toldo et al., 2009; de Vos et al., 2017; Mabray et al., 2020), and to cause an annual hemorrhage rate of 2.1%, with the associated severe symptoms (Badhiwala et al., 2014). We investigated the formation of cavernous malformations in the spinal cord of the same animals. Here also, multi-lumen mulberry-shaped lesions appeared, starting from 3 months of age, thus showing slower kinetics compared to the brain lesions (Figures 3A–3C). Spinal cord cavernous malformations shared the same pathological characteristics of brain CCM, as they showed acute and chronic hemorrhage, formation of blood clots, and leukocyte infiltration (Figures 3D and 3F).

### Endothelial progenitor cell-specific deletion of *Ccm3* results in splenic phenotype and altered hematopoiesis

**Figure 4:**
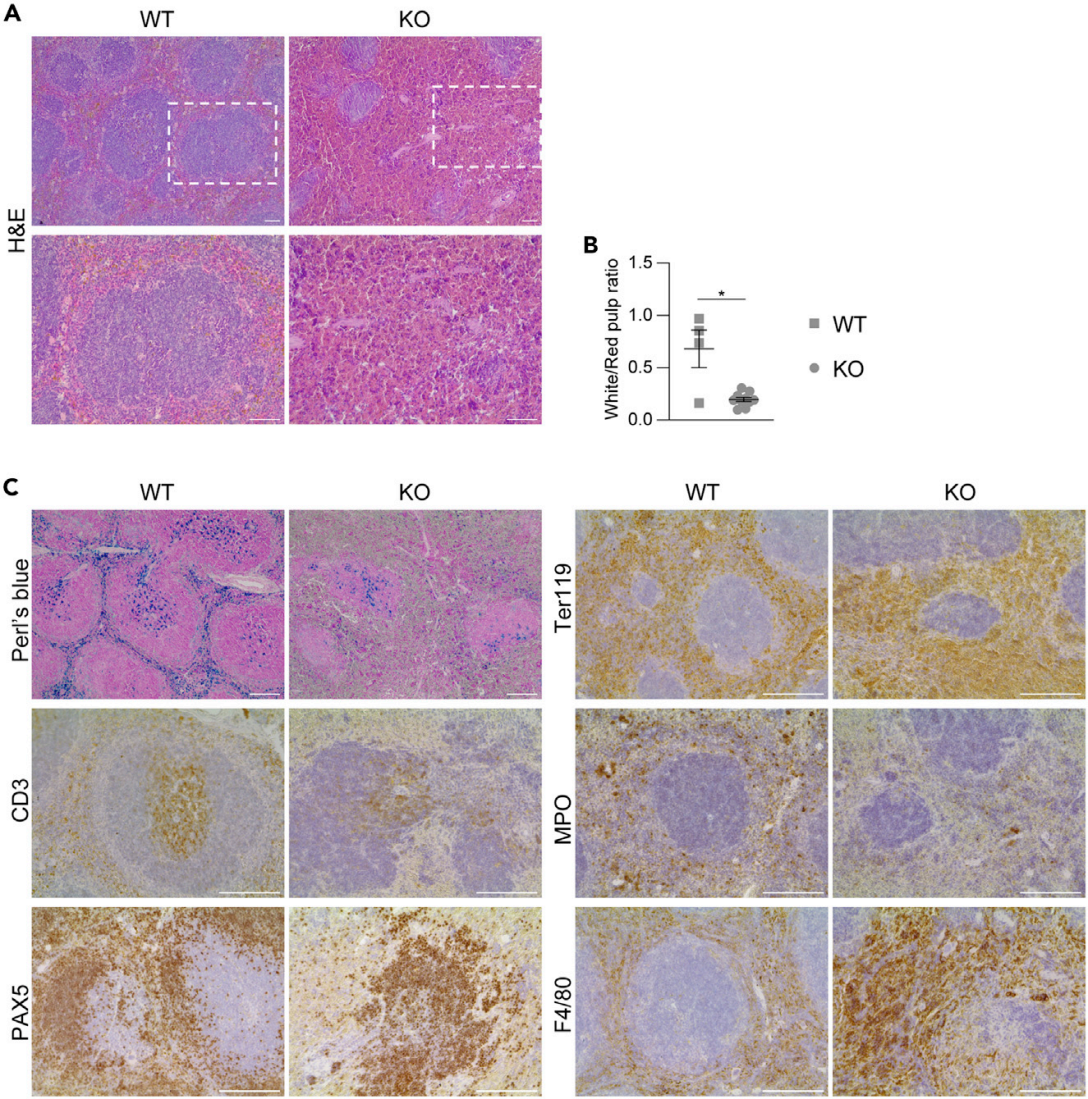
The spleen shows hematopoietic disorders Representative images of the spleen from 3-month-old wild-type (WT) and *Ccm3*^EPCKO^ (KO) mice. Four WT and nine KO mice were analyzed. (A) Representative H&E staining. (B) Quantification of white and red pulp areas. Data are means ± SE. Each dot represents an animal. ∗p < 0.001 (Student’s t-tests). (C) Representative immunostaining for different cell populations. Scale bar: 50 μm (A); 100 μm (C).

**Figure 5:**
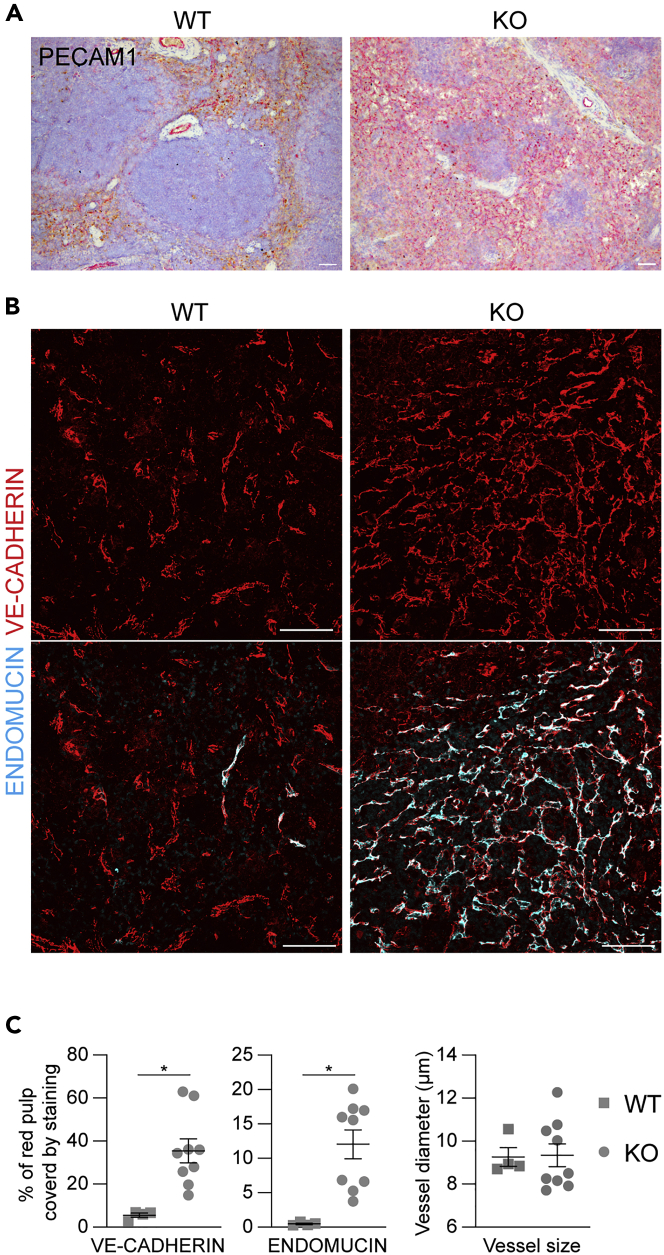
The spleen shows increased vascular density (A and B) Representative images of the spleen stained for the endothelial markers PECAM1 (A), VE-Cadherin (endothelial cells) and Endomucin (sinusoids) (B). Scale bar: 50 μm. (C) Quantification of vascular density and mean vessel diameter. Data are means ± SE. Each dot represents an animal; ∗p < 0.005 (Mann-Whitney tests). Four WT and nine KO 3-month-old mice were analyzed.

To determine whether other organs were affected by deletion of *Ccm3* in the endothelial progenitors, the liver, heart, lungs, kidneys, spleen, and bone marrow of these mice were analyzed at 3 months of age, when the phenotype in the brain became very severe. In the histopathological analysis, no pathological phenotype was seen for the liver, lungs, kidneys, and heart (Figure S5), whereas the spleen showed altered organization of the red and white pulp (Figure 4), but with no obvious splenomegaly. In particular there was consistent regression of the white pulp, with compensatory hyperplasia of the red pulp and concomitant disruption of the marginal zone (Figures 4A and 4B). The white pulp showed overall contraction of the CD3^+^ T-cell areas, and reduced and disorganized B-cell compartment, as revealed by PAX5 staining. The expanded red pulp had the characteristics of a hemorrhagic phenotype, with increased numbers of Ter119-positive erythroid cells, paralleled by decreased numbers of myeloperoxidase-positive myeloid elements. The red pulp also showed diffuse increase of F4/80-positive macrophages (Figure 4C). This phenotype suggested an imbalance in splenic hematopoiesis, which was shifted toward the erythroid components to the detriment of the myelopoiesis. The vasculature of the spleen was dramatically affected, with increased capillary densities mainly in the setting of the red pulp vascular network, which, however, did not show any altered morphology (i.e., no increased lumen size or cavernoma-like structures) (Figure 5).

*In situ* immunophenotypical analyses of the bone marrow hematopoietic populations did not highlight major changes in the density and distribution of the major hematopoietic lineages including megakaryocytes (CD41^+^), macrophages (F4/80^+^), myeloid cells (MPO^+^), and erythroid precursors (Ter-119^+^) (Figures S6 and S7A). The absence of major changes in the hematopoietic phenotype was consistent with a generally preserved vascular architecture in the bone marrow of *Ccm3*^EPCKO^ mice (Figure S8). However, the bone marrow showed a decrease in the overall frequency of Pax5^+^ B-lymphoid elements, in the absence of modifications in CD3^+^ T-cells (Figure S7A). This result suggests that central defects in the support to lymphopoiesis, a process known to be tightly regulated by vascular stromal dynamics (Zehentmeier and Pereira, 2019), could be part of the observed phenotype. The overall hematopoietic phenotype was associated, in the peripheral blood, with increased numbers of circulating leukocytes, which did not show any altered morphology (Figure S7B and S7C). There was no alteration of the vasculature in the other organs, such as the liver (Figure S9), which, at this stage, indicated the spleen as the only peripheral organ where the vasculature was affected.

During the analysis of the 6-month-old mice, additional pathological phenotypes were recorded. Two out of the three mice had hemorrhagic and swollen testes. Histological analysis showed capillary ectasia and congestion, interstitial edema, and hemorrhage associated with disorganization of the germ cells of the seminiferous tubules. The histological picture was reminiscent of cavernous hemangioma of the testis (although the distribution of the ectatic vessels was not properly confined to a tumor-like lesion) (Figure S10A). Very recently, the same phenotype was reported in *Cdh5-Cre*^ERT2^/*Ccm1*^fl/fl^ mice of the same age, which had undergone recombination at 2 months of age. For these mice, none of the other organs, including the brain, showed any vascular abnormalities (Ren et al., 2021). One animal with abnormal testes also developed a tumor mass in the abdominal cavity (Figure S10B).

### Propranolol neither substantially induces regression of CCM lesions nor ameliorates the pathological phenotype

**Figure 6:**
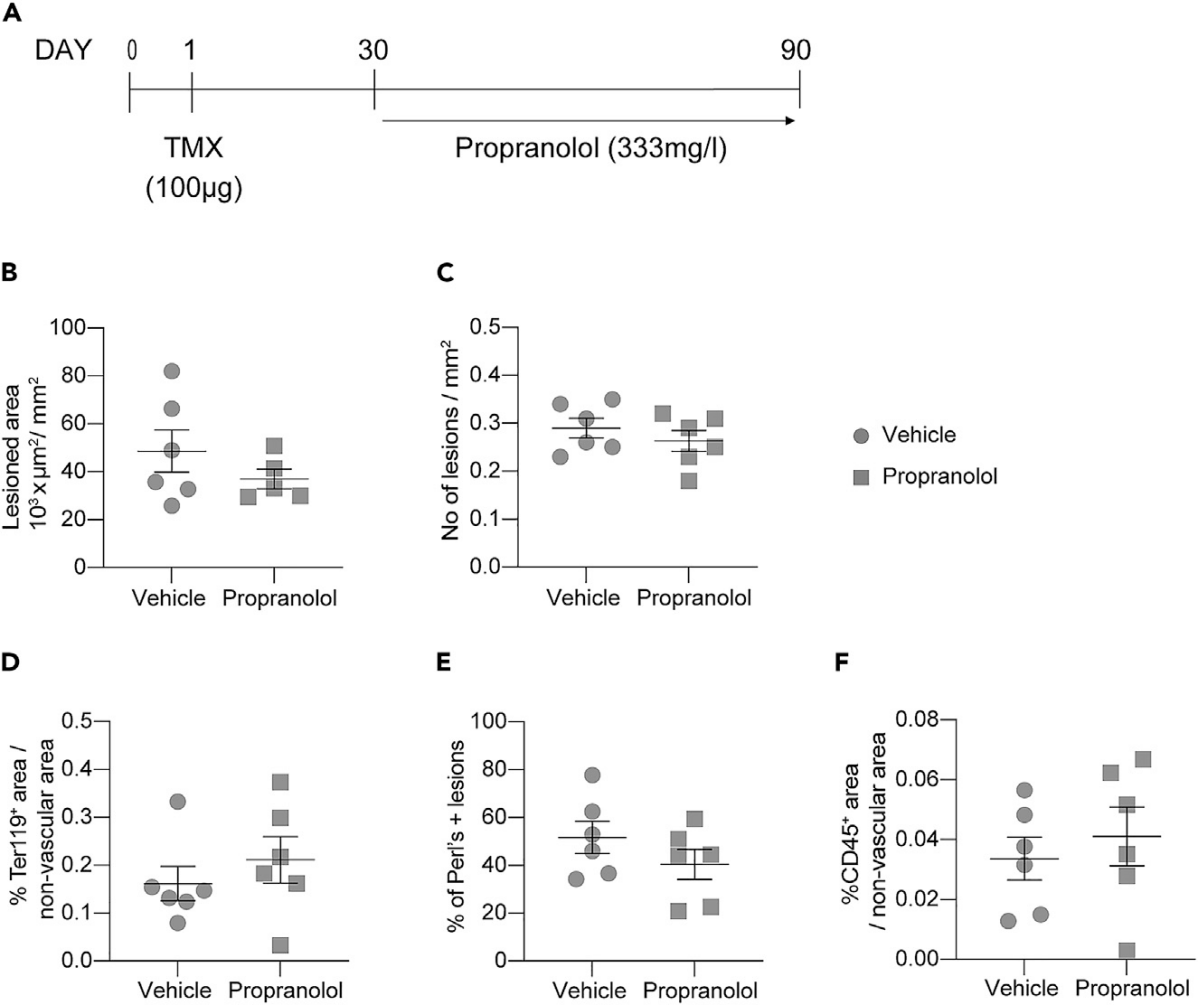
Propranolol administration did not ameliorate the pathological phenotype (A–C) Procr^CreERT2−IRES-td-Tomato/+/^Ccm3^f/f^ were treated with 333 mg/L of propranolol dissolved in the drinking water from one to three months of age. Quantification of (B) total lesioned area and (C) number of lesions in the brain. (D) Quantification of erythrocytes extravasation expressed as percentage of Ter119-positive area outside the vascular area. (E) Quantification of chronic hemorrhage expressed as percentage of mice with at least one field positive for Perls' Prussian stain. (F) Quantification of leukocytes extravasation expressed as percentage of CD45-positive area on nonvascular area. For all the plots, data are means ± SE. Each dot represents an animal; p > 0.05 (Student’s t test).

Propranolol, a nonselective beta-blocker, is a promising drug to treat CCM which entered clinical trials on patients with familial CCM (Treat_CCM) (Lanfranconi et al., 2020). In parallel, chronic treatment with propranolol has shown efficacy in inhibiting lesion formation in three different murine models of *Ccm3* KO (Li, Marchuk, and Awad, no date; Oldenburg et al., 2021). Here, we wanted to test whether propranolol was also effective in reducing lesions already established, rather than in the formation of new ones. With this aim, we treated *Ccm3*^EPCKO^ mice with propranolol in the drinking water for two months starting from one month of age, when CCM lesions appear (Figure 6A). Although propranolol showed a slight reduction of lesion size, we did not observe a statistically significant reduction of lesion burden. Moreover, neither acute and chronic hemorrhage nor inflammatory infiltrates were affected by treatment with propranolol (Figures 6B–6F).

## Discussion

The murine model of CCM characterized here presents pathological characteristics that closely resemble those of human disease. After postnatal deletion of *Ccm3*, the animals develop small CCM lesions, as early as P8, and mainly in the cerebellum. These lesions increase in size and number over time, and they start to form throughout the CNS, with a distribution of around 70%–75% in the cerebrum at 3 months of age; this is in line with the distribution of lesions in familial patients, which is reported to be around 70%–80% in the cerebrum (Brunereau et al., 2000; Labauge et al., 2001; Dalyai et al., 2011). We have also reported and characterized here the formation of multi-lumen mulberry-shaped lesions in the spinal cord with full penetrance. This finding is of major importance, as cavernomas in the spinal cord have an estimated prevalence of 23%–72% in familial patients (Cohen-Gadol et al., 2006; Toldo et al., 2009; de Vos et al., 2017; Mabray et al., 2020), and they cause hemorrhages with an annual rate of 2.1% (Badhiwala et al., 2014). These are associated with major symptoms, which span from muscular weakness (60.5%), through tingling and numbness (57.8%) and pain (33.8%), to bladder and/or bowel impairment (23.6%), and respiratory distress (0.5%). Formation of spinal cord cavernomas is limited to anecdotal reports in existing murine models (Detter et al., 2020; Lopez-Ramirez et al., 2021) and lacks a comprehensive characterization of the features, including the kinetics of onset and the presence of hemorrhage and inflammatory infiltrates. Therefore, this model provides a system for more detailed studies into the characteristics of spinal cord cavernous malformations, and to find drugs that can ameliorate their natural history.

Acute hemorrhage in CCM is the first medical emergency, and can result in most of the clinically relevant symptoms. Therefore, great efforts are being made to find pharmacological treatments that might prevent hemorrhages from one side, and attenuate the parenchymal damage from the other. Despite this, at present, effective medical treatment is still lacking, in part because of the lack of a reliable murine model for hemorrhage in CCM. Here, erythrocyte extravasation and nonheme iron deposition showed that these *Ccm3*^EPCKO^ mice spontaneously develop both chronic and acute hemorrhages.

The *Ccm3*^EPCKO^ model also shows infiltration of immune cells into the lesion site, and increased levels of circulating lymphocytes, as already shown in human patients and mice. Moreover, we describe here a vascular phenotype in the spleen that is characterized by increased vascular density, in particular of the sinusoids of the red pulp. This vascular phenotype is accompanied by imbalanced hemopoiesis, which was shifted toward the erythroid line more than the myeloid and lymphoid lines. Whether this hemopoietic defect originates from the bone marrow or from the spleen, and whether it is correlated with the severity of the brain phenotype, needs more investigation.

The model presented here was generated by restricting the deletion of *Ccm3* to EPC. We have previously shown, in murine models, that EPC play a key role in the initial steps of cavernoma formation and that they are increased within the lesions (Malinverno et al., 2019; Orsenigo et al., 2020). Importantly, endothelial cells lining human cavernomas express much higher levels of PROCR, if compared to normal vessels of healthy donors (Lopez-Ramirez et al., 2019).

A major drawback of acute models of CCM developed over the last two decades is their relatively fast kinetics. Indeed, they develop lesions very soon after birth, and these become lethal at around 2 weeks–4 weeks of age (Boulday et al., 2011; Maddaluno et al., 2013; Bravi et al., 2015). This represents a big limitation when testing the efficacy of drugs, particularly if they are aimed at reducing established lesions. Instead, the *Ccm3*^EPCKO^ mice developed CCM lesions more slowly, with larger lesions appearing at around 1 month of age and mice surviving beyond 6 months. This makes the model suitable for dissection of the molecular mechanisms that underlie CCM, as well as to determine the efficacies of new drugs that can reduce the lesion burden.

During the preparation of this study, other groups developed chronic models of CCMs by restricting the deletion of either the *Ccm3* or *Ccm2* gene to the endothelial cells of the brain microcirculation (Cardoso et al., 2020; Detter et al., 2020; Zhou et al., 2021). These brain-specific CCM models also develop lesions with slow kinetics and chronic hemorrhage. In these other models of CCM, the phenotype develops in response to events that take place only in the brain, and it is devoid of important systemic components. These components of the disease, such as the role of the microbiome (Tang et al., 2017) and the gut-brain axis (Tang et al., 2019), of systemic inflammation, and of the immune response, have been shown to be important to determine the severity of the CCM phenotype. Therefore, this restriction of the deletion of CCM genes to the brain leads to increased survival of animals that develop CCM lesions, which, however, might lack important characteristics, such as acute hemorrhage.

As proof of principle, we tested the effect of Propranolol on *Ccm3*^EPCKO^ mice. Propranolol is a nonselective beta-blocker which is widely used to treat hypertension (Al-Majed et al., 2017) and other vascular diseases such as infantile hemangioma (Léauté-Labrèze et al., 2015). Propranolol thus shows promise as a treatment option for CCM, and we have started a randomized controlled clinical trial to test the efficacy of propranolol treatment on patients with familial CCM (Treat_CCM) (Lanfranconi et al., 2020). In our previous preclinical study (Oldenburg et al., 2021), we treated *Cdh5*-CreER^T2^/*Ccm3* mice for one month starting after birth by giving propranolol in the drinking water. In this way, the pups received the drug through the mother’s milk until weaned (P21) and then directly via the water. The treatment with Propranolol inhibited the onset and progression of the disease and resulted in fewer and smaller lesions. Here, to test whether the propranolol was effective on lesions already established, we administered via the drinking water the same concentration of drug to the mice from one month of age for two months. With this protocol of treatment propranolol did not ameliorate any of the pathological features of CCM i.e., lesion burden, acute and chronic hemorrhage, or inflammatory infiltrate. The lack of curative effect of propranolol under these conditions can be attributed i) to the dose which, although being effective in pups, is too low for juveniles or adults, or ii) to the intrinsic mechanism of propranolol that has different effect on lesion genesis and maturation, as already proposed (Li, Marchuk, and Awad, no date; Oldenburg et al., 2021). However, the extended viability of the *Ccm3*^EPCKO^ model will allow researchers to optimize more accurately the timing and dose of propranolol administration to find the best conditions of treatment.

### Limitations of the study

Despite the advantages of the *Ccm3*^EPCKO^ model discussed above, our study has limitations.

1) We have not been able to make a direct comparison with other slow progression models recently developed by other groups, in particular the brain endothelium-restricted models. This would be necessary to clarify advantages and disadvantages of each model. We hope that such a comparison will be possible in the future, in order to provide the CCM research community with a common platform of useful murine models to be used for mechanistic studies and drug screening.
2) *Ccm3*^EPCKO^ mice develop CCM lesions only if tamoxifen is administered at neonatal stage, whereas no lesion forms when tamoxifen is given to adult mice. This is true and well described for all other murine models of CCM developed so far (Boulday et al., 2011; Detter et al., 2020; Ren et al., 2021; Zhou et al., 2021), and represents a general limitation of these models.

### Conclusion

In conclusion, this murine model of CCM generated by deletion of *Ccm3* specifically in endothelial progenitors resembles all of the major features of human disease. Here, multi-lumen mulberry shaped mature lesions develop throughout the CNS, including the spinal cord, and these are characterized by immune cell infiltrates, chronic bleeding, and more importantly, acute hemorrhage. Therefore, this mouse model of CCM provides a new and useful preclinical model to test drugs, with a particular focus on regression or stabilization of existing cavernomas that are prone to bleeding or have already shown bleeding. This should foster the identification and development of new pharmacological therapies to treat intracerebral hemorrhage, the unmet medical emergency for patients with CCM.

## STAR★Methods

### Key resources table

**Table:** 

REAGENT or RESOURCE	SOURCE	IDENTIFIER
**Antibodies**		

Armenian hamster anti-Cd31 monoclonal	Merck Millipore	Cat# MAB1398Z, RRID:AB_94207
rat anti-mouse Ter119	BD Biosciences	Cat# 553671, RRID:AB_394984
rat anti-CD45R	BD Pharmigen	Cat# 550286, RRID:AB_393581
rabbit anti-collagen IV polyclonal	Bio-Rad	Cat# 2150-1470, RRID:AB_2082660
goat anti-VE-cadherin	R&D	Cat# AF1002, RRID:AB_2077789
rat anti-endomucin	Abcam	Cat# ab106100, RRID:AB_10859306
rabbit anti-Cd41	Abcam	Cat# ab181582, RRID:AB_2904562
rabbit anti-F4/80	Cell Signaling	Cat# 70076, RRID:AB_2799771
rabbit anti-IBA1	Fujifilm Wako Shibayagi	Cat# 019-19741, RRID:AB_839504
rabbit anti-Cd31	Abcam	Cat# ab28364, RRID:AB_726362
rabbit anti-myeloperoxidase	Abcam	Cat# ab208670, RRID:AB_2864724
rabbit anti-Cd3	Cell Signaling	Cat# 99940, RRID:AB_2755035
rabbit anti-Pax5	Abcam	Cat# ab109443, RRID:AB_10862070
goat anti-Armenian hamster Cy3-conjugated	Jackson Immuno Research	Cat# 127-165-160, RRID:AB_2338989
donkey anti rat Alexa Fluor 488-conjugated	Jackson Immuno Research	Cat# 712-545-153, RRID:AB_2340684
ultrapolymer goat anti-rabbit IgG conjugated to HRP (H&L)	Immunoreagents, Inc.	Cat# GARHRP-015, RRID:AB_2904563
rabbit anti goat biotinylated	Vector Laboratories	Cat# BA-5000, RRID:AB_2336126

**Chemicals, peptides, and recombinant proteins**		

Tamoxifen	Sigma-Aldrich	Cat# T5648
2,2,2-Tribromoethanol	Sigma-Aldrich	Cat# T48402
30% hydrogen peroxide solution	Sigma-Aldrich	Cat# H1009
Eukitt mounting medium	Sigma-Aldrich	Cat# 03989
Paraformaldehyde	Sigma-Aldrich	Cat# P6148
4% formaldehyde	VWR	Cat# 9713
MicroDec	Diapath	Cat# D0052
Vectashield	Vector Laboratories	Cat# H-1200
tert-Amyl alcohol	Sigma-Aldrich	Cat# 8.06193

**Critical commercial assays**		

Perls kit reagents	Bio-Optica	Cat# 04-180807
DAB kits	Vector Laboratories	Cat# SK-4100-KI01
Vulcan fast red kits	Biocare	Cat# FR805

**Experimental models: Organisms/strains**		

*Procr*^CreERT2-IRES-tdTomato/+^	Dr. Ariel Zeng, University of Shanghai	N/A
*Ccm3 floxed*	Prof. Elisabetta Dejana, IFOM	N/A
Cdh5-CreRT2	Dr. Ralf Adams, UK Cancer research	N/A

**Software and algorithms**		

Fiji - Imagej	Nat Methods. 2012 Jun 28; 9(7): https://doi.org/10.1038/nmeth.2019.	https://imagej.net/software/fiji/
GraphPad Prism 9	GraphPad Software, Inc.	https://www.graphpad.com/support/faq/prism-900-release-notes/

### Resource availability

#### Lead contact

Further information and requests for resources and reagents should be directed to and will be fulfilled by the lead contact Matteo Malinverno (matteo.malinverno@ifom.eu)

#### Materials availability

The animal models used in the study are available upon request from the Lead Contact with a material transfer agreement.

### Experimental model and subject details

#### Animal studies

All of the procedures with the mice were performed in agreement with the Institutional Animal Care and Use Committee (IACUC) of FIRC Institute of Molecular Oncology, in compliance with the guidelines established in the Principles of Laboratory Animal Care (Directive 86/609/EEC) and as approved by the Italian Ministry of Health.

The animals housed under standard conditions and were fed with a regular chow diet *ad libitum*.

The *Cdh5*(PAC)-Cre-ER^T2^/*Ccm3*^f/f^ mice in which *Ccm3*^f/f^ mice with exons 4–5 of the *Ccm3* gene flanked by loxP sites (Taconic Artemis GmbH) were bred with *Cdh5*(PAC)-Cre-ER^T2^mice to obtain endothelial-specific and tamoxifen-inducible loss of function of the *Ccm3* gene, as previously described (Bravi et al., 2015). The *Procr*^CreERT2-IRES-tdTomato/+^/*Ccm3*^f/f^ mouse strain (here named *Ccm3*^EPCKO^) was generated previously by Malinverno et al. (2019) (Malinverno et al., 2019). This model combines the *Ccm3* gene floxed (*Ccm3*^f/f^) with the inducible form of the Cre recombinase driven by the *Procr* promoter (*Procr*^CreERT2-IRES-tdTomato/+^). The administration of tamoxifen induces the recombination, and therefore the deletion of the *Ccm3* gene, specifically in *Procr*-positive endothelial progenitor cells.

*Ccm3*^ECKO^ strain is pure C57/BL6, while *Ccm3*^EPCKO^ strain is on a mixed background. Littermates of both sexes were used in this study, and analyzed at the indicated age: 8 days, 1, 2, 3, 4 and 6 months. For the propranolol study, after weaning, animals of both sexes were randomized and assigned to either vehicle or propranolol group.

### Method details

#### Tamoxifen injection

Tamoxifen was dissolved in ethanol to 20 mg/mL and then diluted in corn oil to the final concentration of either 2 or 0.1 mg/mL. Mice received a single dose of 100 μg (*Ccm3*^EPCKO^) or 5 μg (*Ccm3*^ECKO^) tamoxifen (final volume 50μL) by oral gavage, using a plastic feeding tube (22 ga x 25 mm) at either 1, 2 or 3 days after birth (P1, P2, P3), as indicated in the text.

#### Immunohistochemistry and imaging

The mouse tissues were collected from mice anesthetized with a lethal dose (20 mg/kg) of avertin (2,2,2-tribromoethyl alcohol in tert-amyl alcohol) following tissue perfusion with phosphate-buffered saline (PBS) followed by 4% paraformaldehyde. The brains were removed from the skulls and post-fixed in 4% paraformaldehyde overnight at 4 °C. The spinal cords were extracted and post-fixed in 4% paraformaldehyde overnight, with the whole vertebral column and extruded using hydraulic pressure. The spleen, kidneys, liver, and heart were dissected out and post-fixed in 4% formaldehyde overnight at 4 °C, and then processed with the standard paraffin-embedding procedure. Bone marrow was extracted, decalcified in an EDTA-based solution (Microdec), and processed with the paraffin-embedding procedure. For paraffine embedding, the whole organs were processed by a Diapath automatic processor as follow. Tissues were dehydrated through 70% (60 minutes), 2 change of 95% ( 90 minutes each) , and 3 change of 99% ( 60 minutes each) ethanol, cleared through 3 changes of xylene ( 90 minutes each), and finally immersed in 3 changes of paraffin, 1 hour each. Samples were embedded in a paraffin block and store at room temperature until ready to section.

For the tissue analysis, the brains and spinal cords were included in 4% low-melting-point agarose, and 100-μm-thick sections were cut (VT1200s vibratome; Leica, Wetzlar, Germany) and stained free-floating. For immunofluorescence, the sections were incubated overnight with the primary antibodies diluted in 5% donkey serum, 2% bovine serum albumin and 0.3% Triton-X100 in PBS at 4 °C. The sections were then washed in PBS and incubated with the appropriate fluorophore-conjugated secondary antibodies (goat anti-Armenian hamster Cy3-conjugated and donkey anti rat Alexa Fluor 488-conjugated) for 4 h at room temperature, then washed and post-fixed with 4% paraformaldehyde and mounted using Vectashield (H-1200; Vector Laboratories).

For immunohistochemistry, the sections were blocked with 0.3% hydrogen peroxide for 10 min at room temperature, then washed in PBS and incubated with the primary antibody diluted in 5% goat normal serum and 0.3% Triton-X100 in PBS at 4 °C. Then the sections were washed in PBS and incubated with HRP-conjugated secondary antibody for 4 h at room temperature. The hemosiderin signal was revealed using Perls’ kit reagents, while the primary antibody was detected using either DAB substrate (SK-4100-KI01; Vector Laboratories) or Vulcan fast red kits (FR805; Biocare), before dehydration and mounting with Eukitt (03989; Sigma-Aldrich).

### Quantification and statistical analysis

#### Quantification of lesions, hemorrhage and inflammatory infiltrates

For lesions quantification, 100-μm thick vibratome sections of brain and spinal cord were prepared and stained for the endothelial marker PECAM1 as described above in the “*Immunohistochemistry and imaging”* section. For each brain, at least 10 sagittal sections of whole-brain were cut, and z-stacks were acquired with a 10*x* objective. After maximal projection, each individual lesion was manually outlined by a blinded operator using the FIJI software (Schindelin et al., 2012). The number and the area of the lesions were then measured by the same software and analyzed using GraphPad Prism 9, as described below.

For the analysis of acute hemorrhage, the sections were stained with Ter119 and under light microscopy 20× images were taken in different areas of the spinal cord and brain. The Ter119 positive area was quantified outside the vessels, which were identified as PECAM1-negative areas, with a mask created with the Fiji software.

For leukocyte extravasation analysis, either the total CD45-positive area or the CD45-positive area outside of the vessels were detected. Then light microscopy 20× images were selected and analyzed with the same system described for acute hemorrhage.

#### Quantification of vascular density

For each sample, a 1-mm^2^ area was acquire at the confocal light microscope using the 20× objective. For the analysis, only the red pulp region was considered, and the VE-Cadherin and endomucin staining were analyzed separately. The same threshold was applied to all of the samples, and then the proportion (%) of the covered area was determined using the *Measure* tool of the ImageJ software.

#### Statistical analysis

All graphical representations and statistical analyses were performed with GraphPad Prism 9. All the analysis were performed by a blinded operator. Sample-size was not estimated *a priori*. For the propranolol study, after weaning, animals of both sexes were randomized and assigned to either vehicle or propranolol group. Outliers were removed using the ROUT method with Q = 1%, and a normal distribution was verified using Shapiro–Wilk tests. For normally and nonnormally distributed datasets, the statistical significance was determined using either one-way analysis of variance (ANOVA) test followed by Tukey’s *post-hoc* analysis, or the Kruskal–Wallis test followed by uncorrected Dunn’s *post-hoc* analysis, respectively. For the analysis of Figures S2B and S2C, only two groups were compared within each time point, i.e., P8 and P60, and unpaired two-tailed Student’s t-tests were applied. Dot-plot were used to show the number of samples for each group, where each dot represents an individual animal; data were plotted as means ±SE, as also indicated in Figure legends.

## Data Availability

• Data reported in this paper will be shared by the lead contact upon request • This paper does not report original code. • Any additional information required to reanalyze the data reported in this paper is available from the lead contact upon request.
