# Supplementary material for: A murine model of cerebral cavernous malformations with acute hemorrhage
Source: iScience. 2022 Feb 18;25(3):103943. doi: 10.1016/j.isci.2022.103943 (PMC8898922; doi:10.1016/j.isci.2022.103943)
Supplement: Document S1. Figures S1–S10 [file mmc1.pdf]

iScience, Volume *25*

Supplemental information

A murine model of cerebral cavernous malformations with acute hemorrhage

Claudio Maderna, Federica Pisati, Claudio Tripodo, Elisabetta Dejana, and Matteo Malinverno

Supplemental figure 1

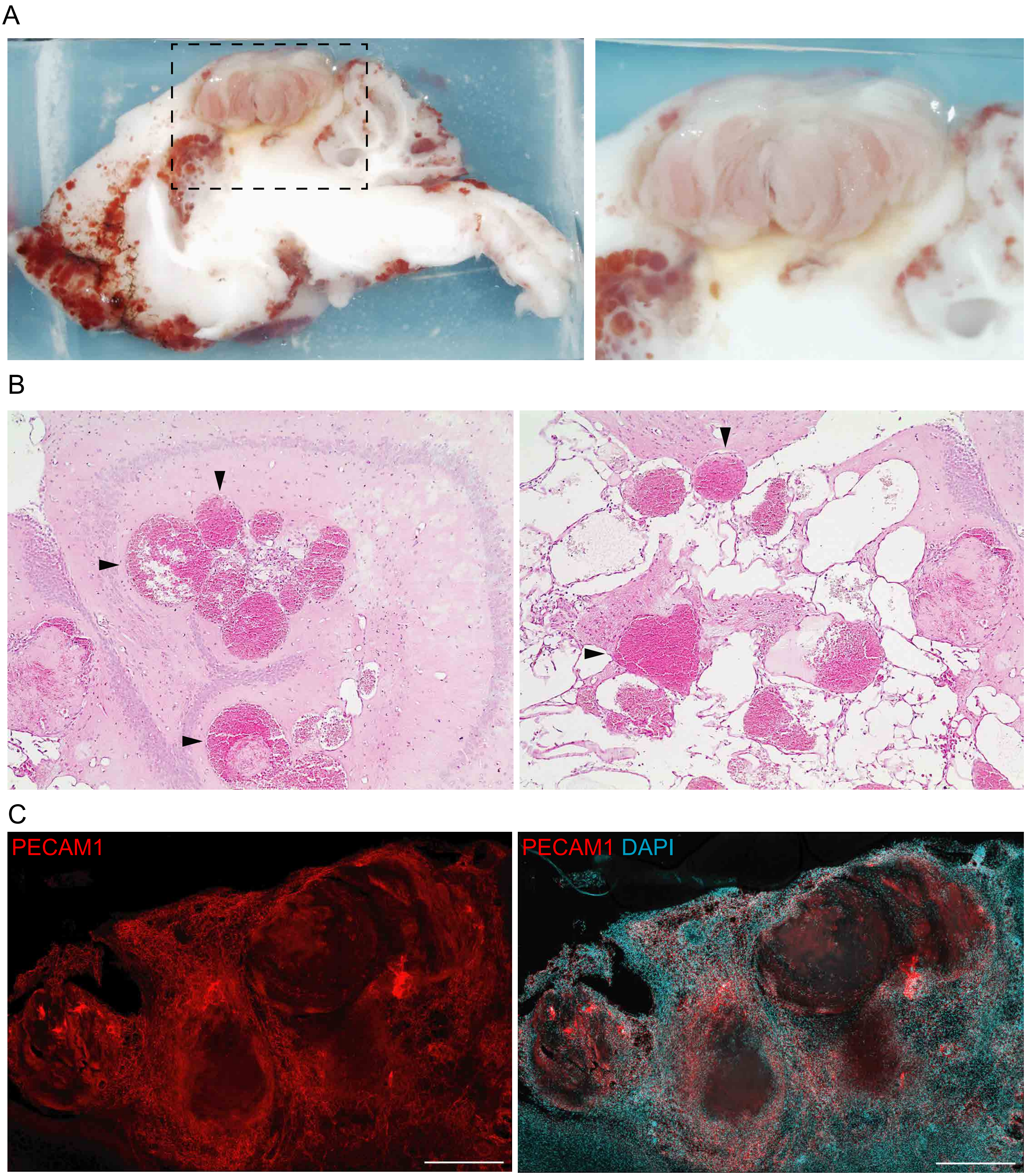

Supplemental figure 1. Giant cavernoma in 6-month-old mouse. Related to Figure 1.
A) Photographs of brain from a 6-month-old *Ccm3*^{EPCKO} mouse showing a giant cavernoma in the cortex. **B)** Relative hematoxylin and eosin staining; arrowheads point to multi-lumen blood-filled lesions. **C)** Confocal microscopy image of the cavernoma stained for PECAM1 and DAPI. Scale bars: 500 μm.

Supplemental figure 2

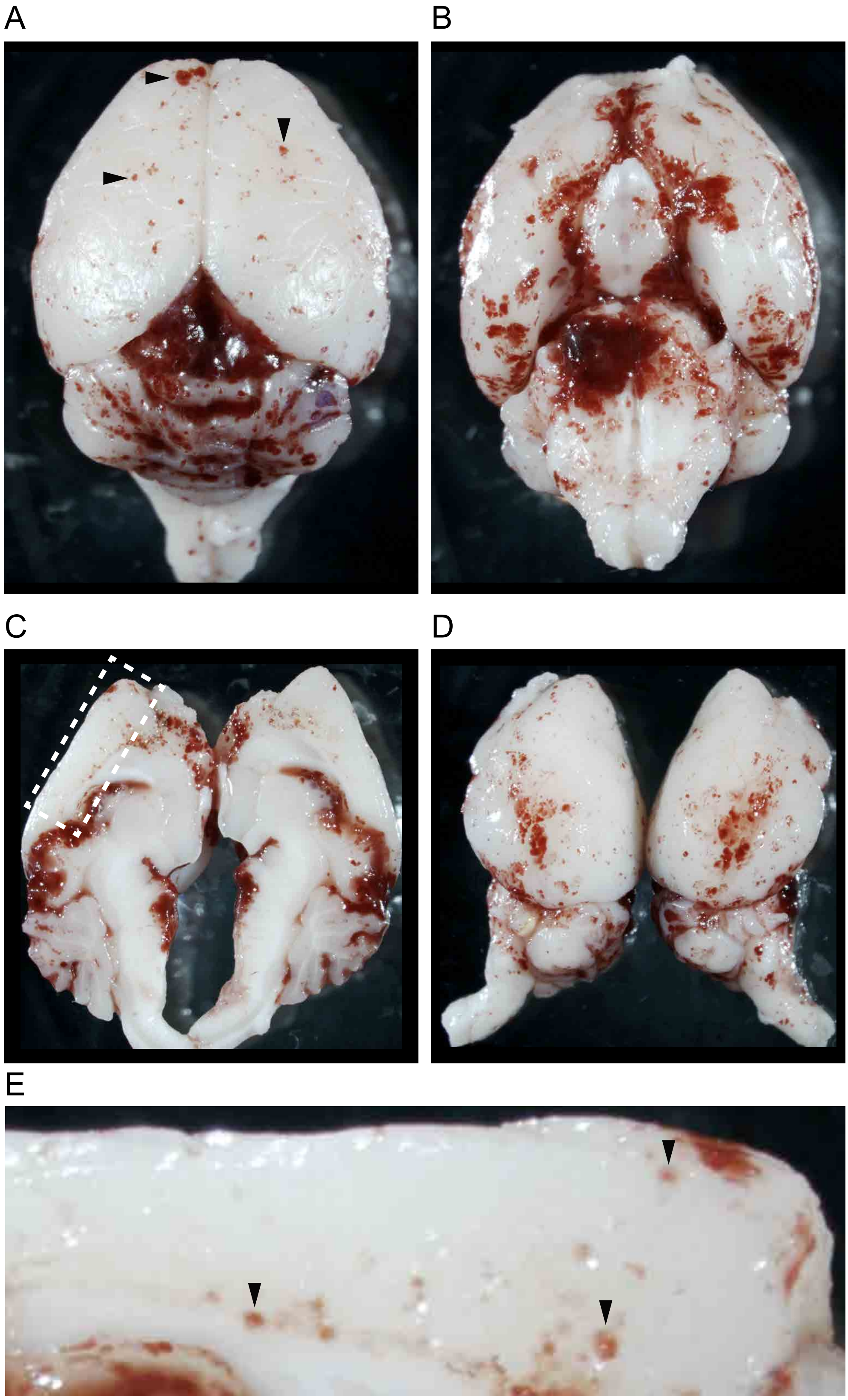

Supplemental figure 2. Lesions form throughout the brain. Related to Figure 1.
Representative photographs of whole brains from mice analyzed at 90 days. Images show: dorsal **A)** and ventral **B)** views of whole brains; midline **C)** and lateral **D)** sagittal surfaces of hemispheres; **E)** magnification from **C)** showing lesions.

Supplemental figure 3

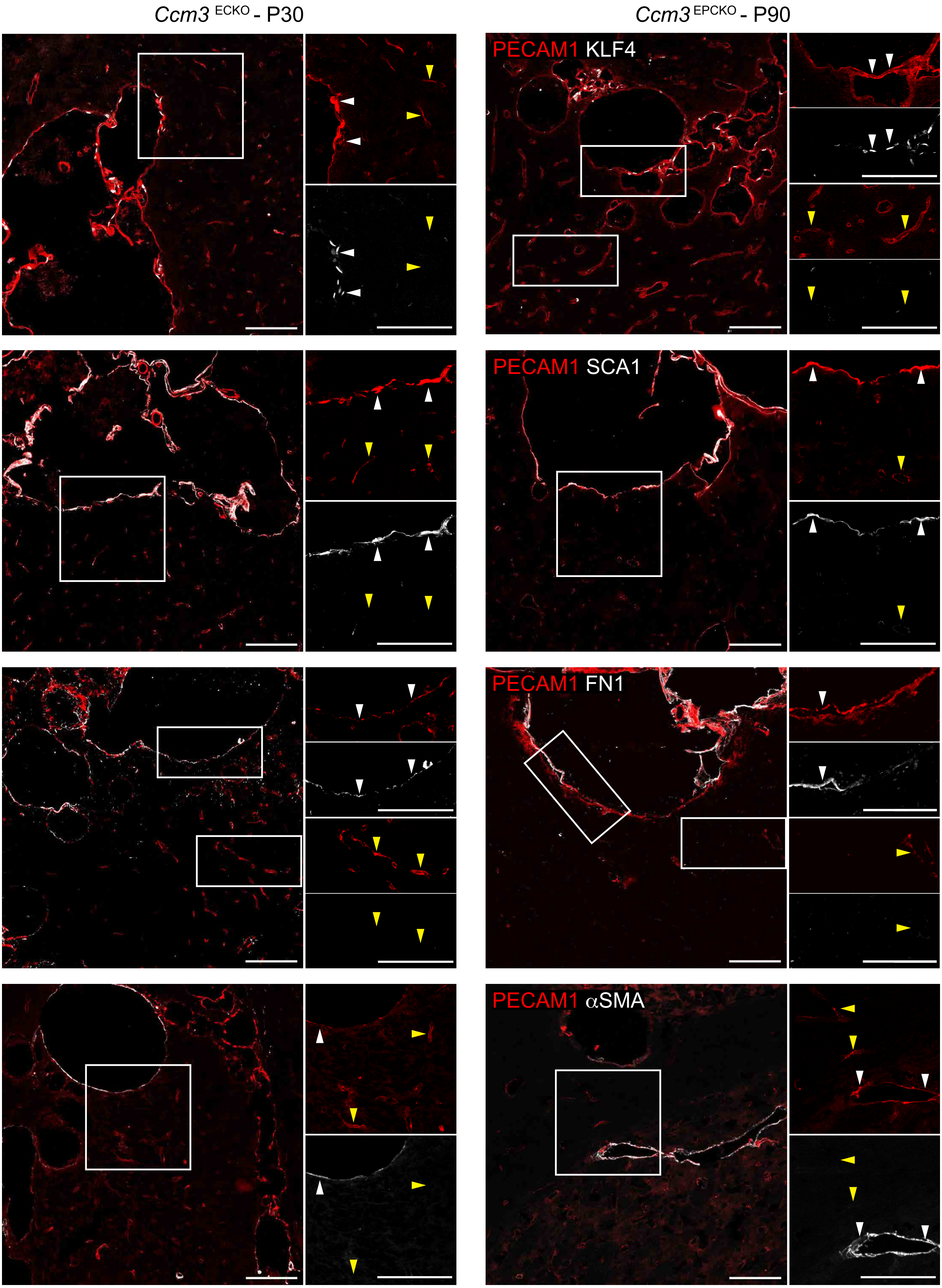

Supplemental figure 3. ECs lining cavernomas express KLF4 and EndMT genes. Related to Figure 1. Representative confocal images showing double staining for PECAM1 as endothelial marker (red) and EndMT genes (gray). Images are taken from brains of *Ccm3*^{ECKO} mice at P30 and of *Ccm3*^{EPCKO} mice at P90. White arrowheads, EC in lesions; yellow arrowheads, EC in normal vessels. Scale bar: 100µm.

Supplemental figure 4

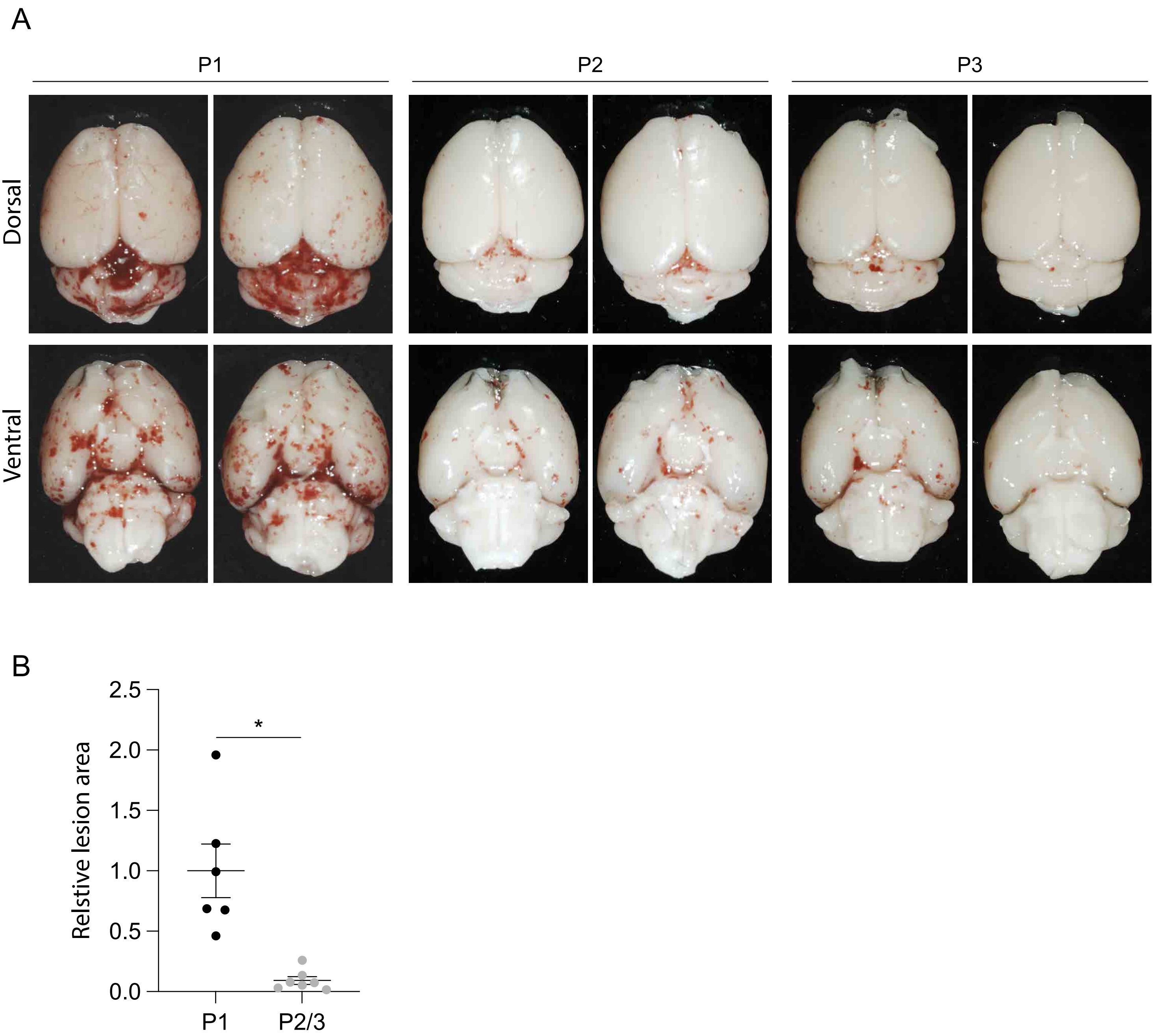

Supplemental figure 4. Delayed tamoxifen administration results in slower disease progression and milder phenotype. Related to Figure 1.
A) Representative photographs of whole brains from mice injected with tamoxifen at either P1, P2 or P3 and analyzed at three months of age. **B)** Quantification of total lesioned area normalized on average area of animals injected at P1. Data are means ±SE; *p <0.01 (Student's t-tests).

Supplemental figure 5

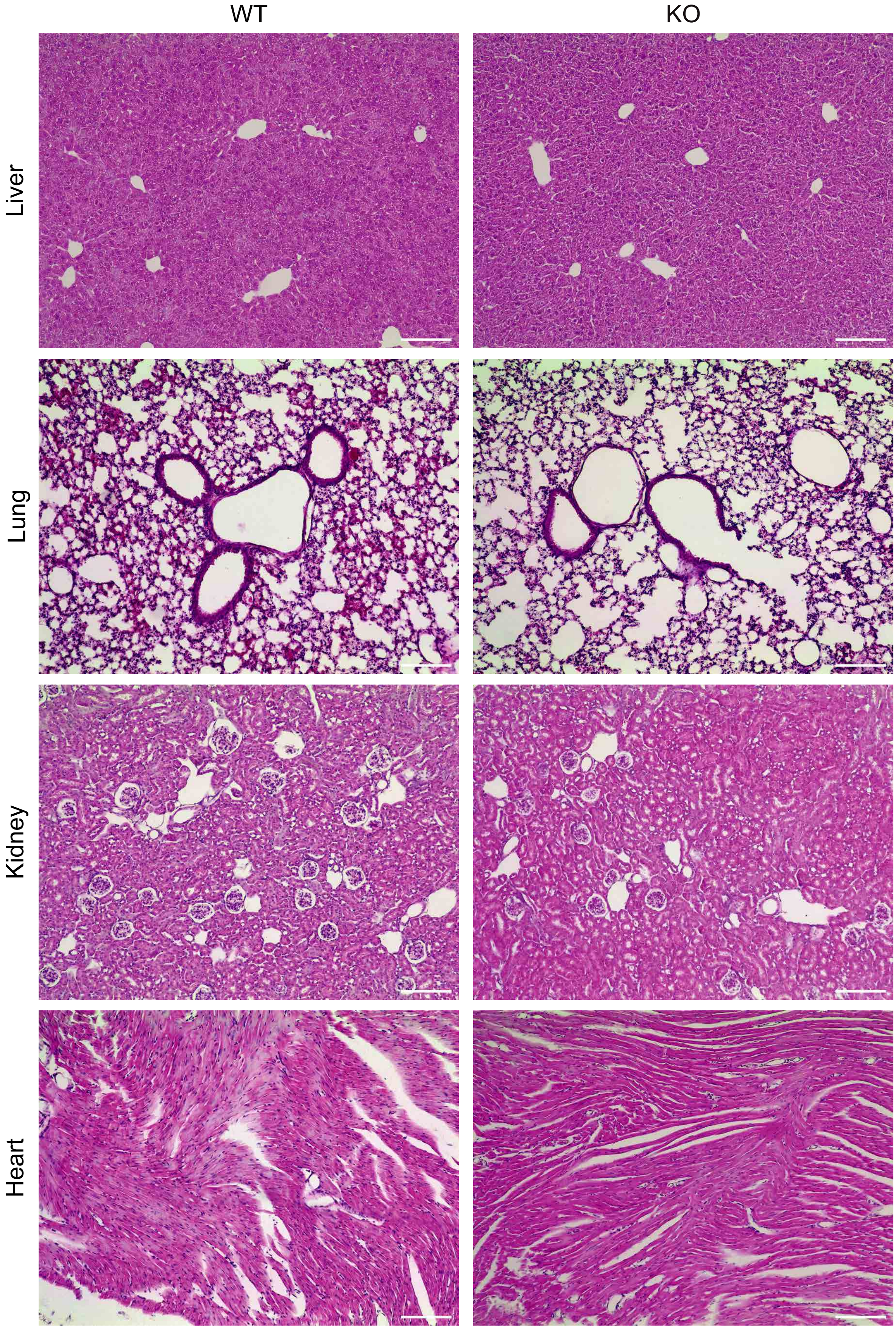

Supplemental figure 5. Histological analysis of peripheral organs. Related to figure 4. Representative hematoxylin and eosin staining of liver, lung, kidney, and heart (as indicated) of 3-month-old wild-type (WT) and *Ccm3*^{EPCKO} mice. Scale bars: 100 μm.

Supplemental figure 6

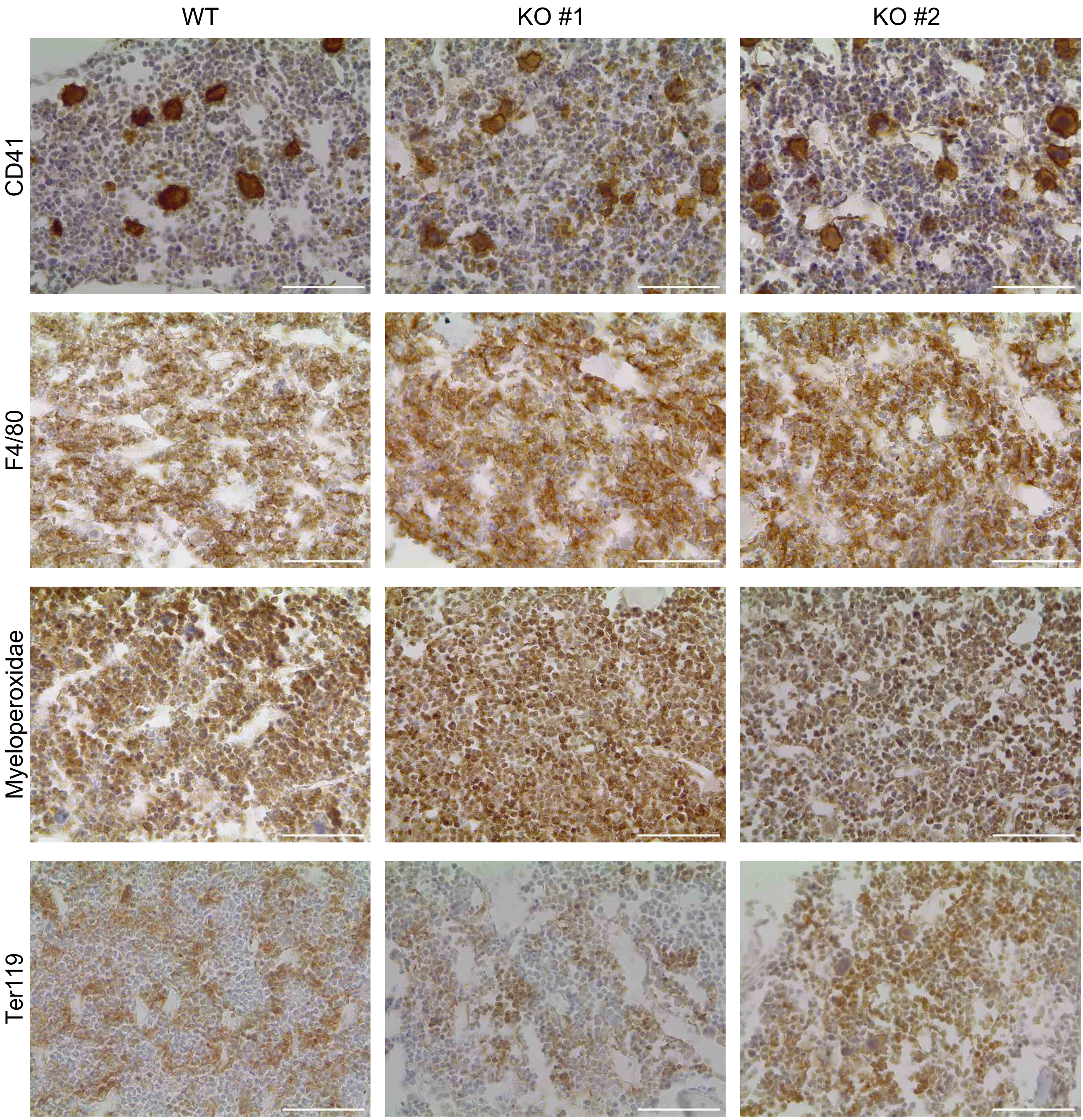

Supplemental figure 6. Bone marrow shows a phenotype similar to the spleen. Related to Figure 4. Representative images of bone marrow of 3-month-old wild-type (WT) and *Ccm3*^{EPCKO} mice stained for CD41 (megakaryocytes), F4/80 (macrophages), myeloperoxidase (myeloid cells), and Ter119 (erythrocytes) (as indicated). A total of four WT and nine knock-out mice were analyzed.

Supplemental figure 7

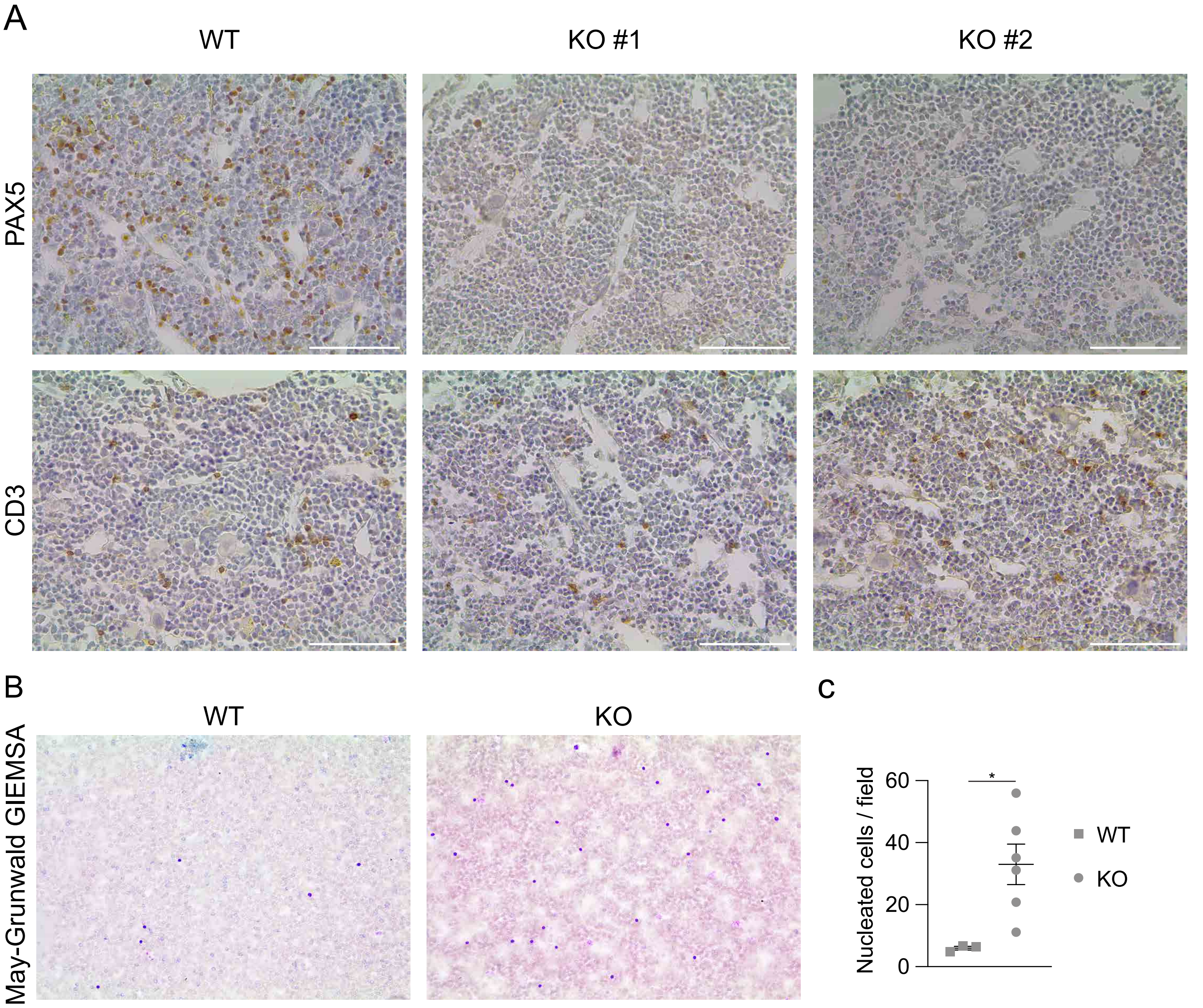

Supplemental figure 7. Bone marrow shows a phenotype similar to the spleen. Related to Figure 4. **A)** Representative images of bone marrow of a 3-month-old wild-type (WT) and *Ccm3*^{EPCKO} mice stained for PAX5 (B-cells) and CD3 (T-cells), as indicated. A total of four WT and nine knock-out mice were analyzed. Scale bars: 50 µm. **B)** Representative images of blood smears with May–Grunwald–Giemsa staining. A total of three WT and seven knock-out mice were analyzed. Scale bar: 100 µm. (c) Quantification of circulating nucleated cells. Data are means ±SE. Each symbol represents an animal; *p <0.03 (Student's t-tests).

Supplemental figure 8

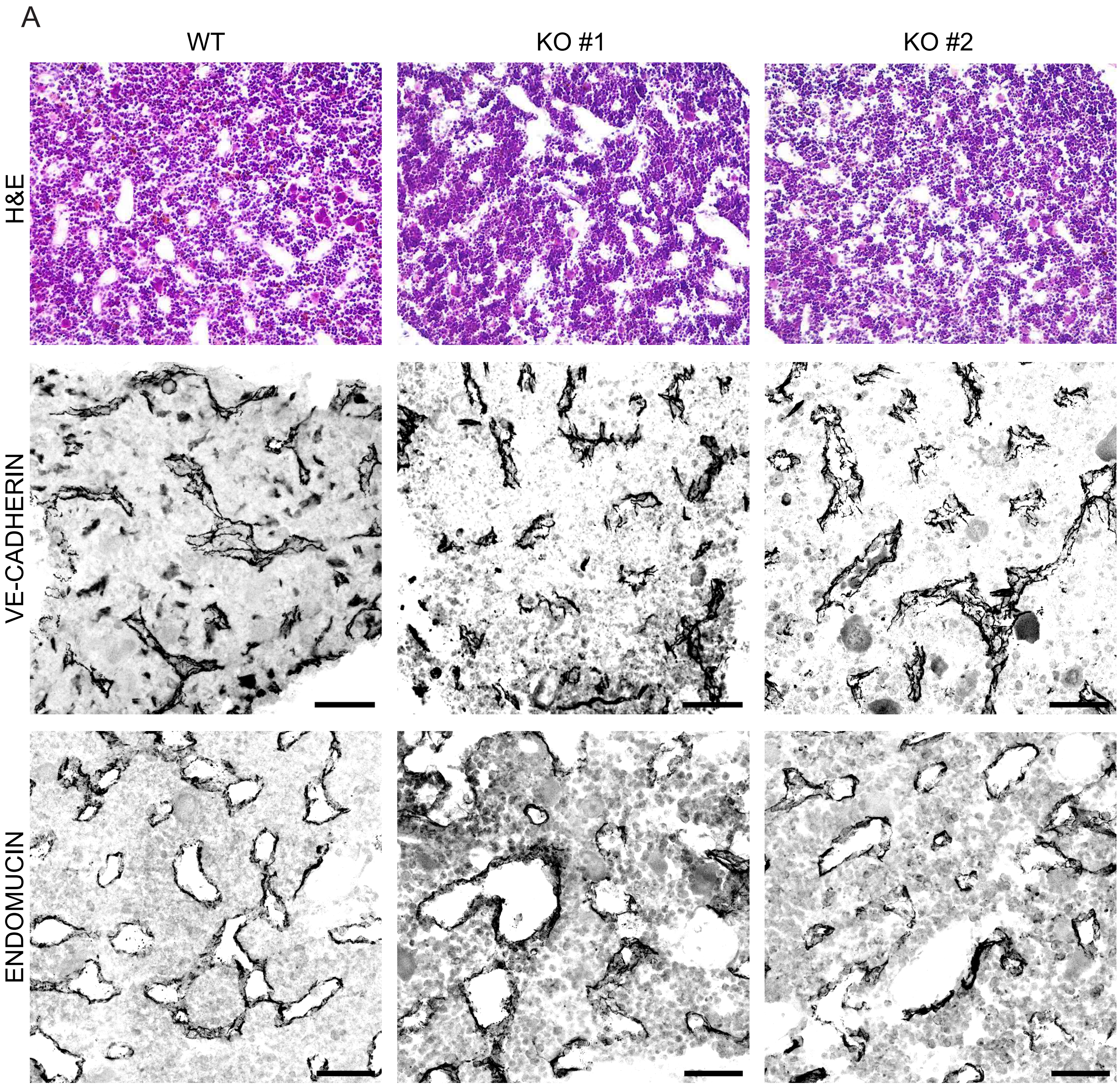

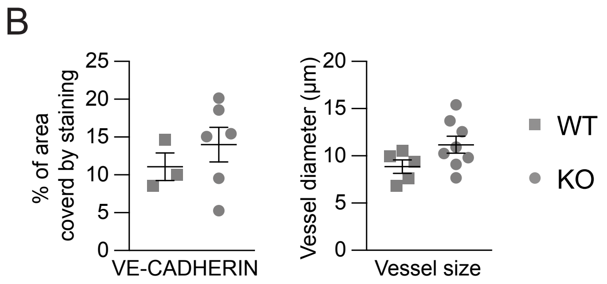

Supplemental figure 8. Bone marrow has a normal vasculature. Relaated to Figure 5.
A) Representative images of bone marrow of a 3-month-old wild-type (WT) and *Ccm3*^{EPCKO} mice stained for hematoxylin and eosin and the endothelial markers VE-cadherin and Endomucin (as indicated). Scale bars: 50 µm. **B)** Quantification of vascular density and mean vessel diameter. Data are means ±SE. Each symbol represents an animal; $p > 0.05$ (Student's t-tests).

Supplemental figure 9

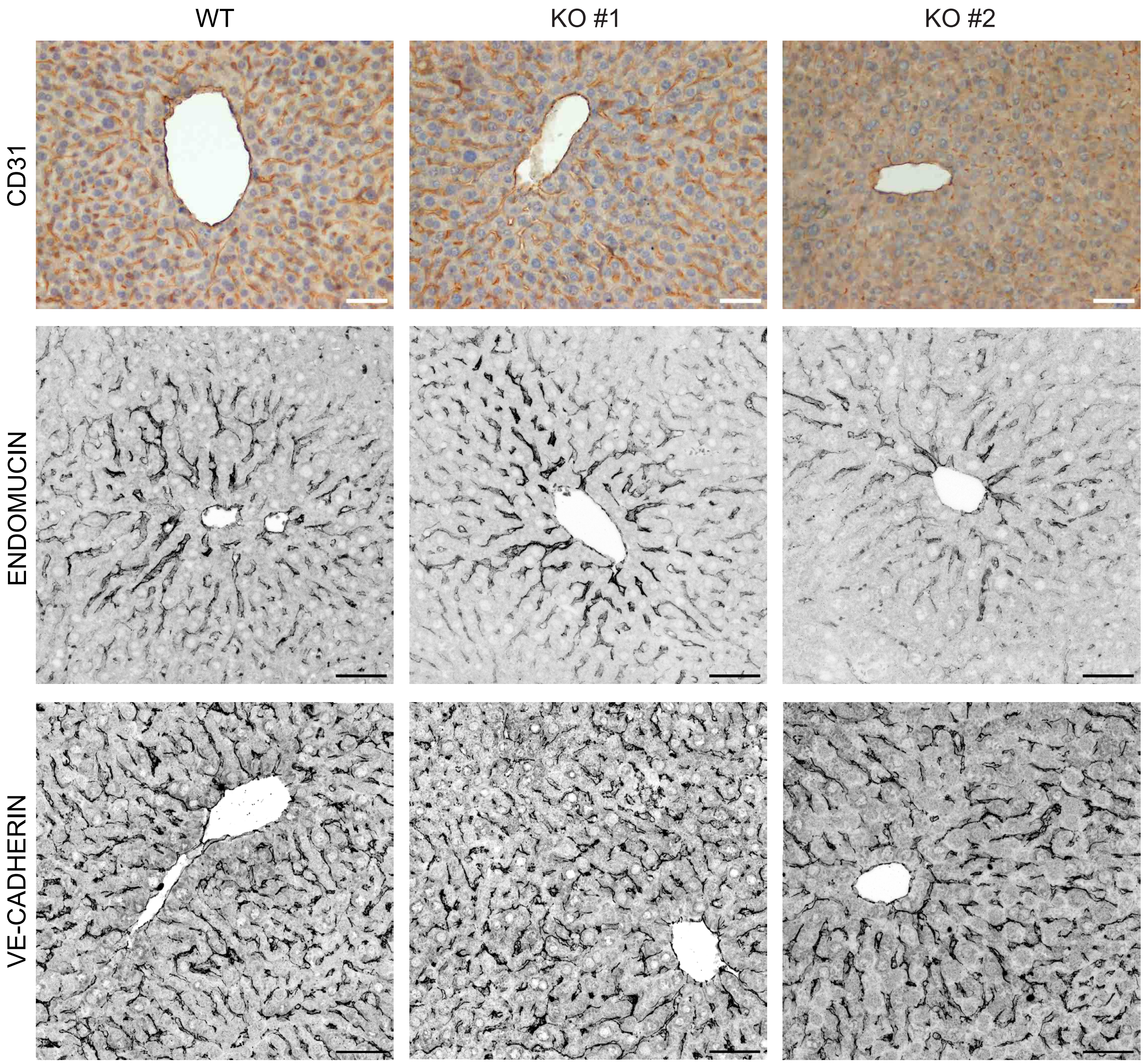

Supplemental figure 9. The liver has a normal vasculature. Related to Figure 5. Representative images of the liver of a 3-month-old wild-type (WT) and *Ccm3*^{EPCKO} mouse stained for hematoxylin and eosin and the endothelial markers VE-cadherin and Endomucin (as indicated). Scale bars: 50 µm.

Supplemental figure 10

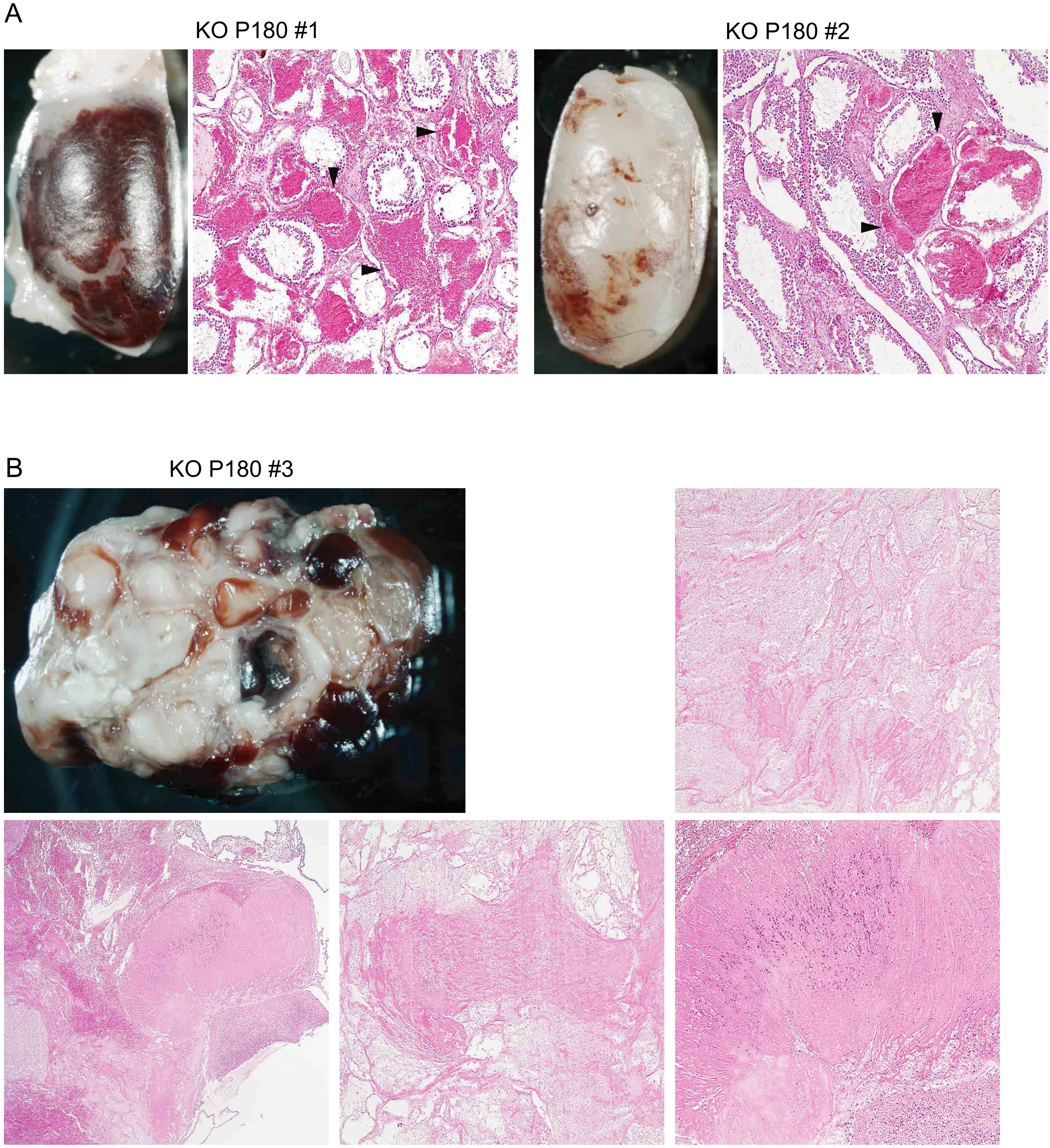

Supplemental figure 10. Other organs affected in 6-month-old mice. Related to Figure 4.
A) Representative photographs of testis from 6-month-old *Ccm3*^{EPCKO} mouse, and relative hematoxylin and eosin staining; arrowhead point to hemorrhage. **B)** Representative photograph of a tumor from the abdominal cavity of a 6-month-old *Ccm3*^{EPCKO} mouse, and relative hematoxylin and eosin staining.
